# Pressure and Temperature Combined With Microbial Supernatant Effectively Inactivate *Bacillus subtilis* Spores

**DOI:** 10.3389/fmicb.2021.642501

**Published:** 2021-05-18

**Authors:** Jingyu Li, Yaxin Sun, Fang Chen, Xiaosong Hu, Li Dong

**Affiliations:** College of Food Science and Nutritional Engineering, National Engineering Research Center for Fruit and Vegetable Processing, Key Laboratory of Fruits and Vegetables Processing, Ministry of Agriculture, Engineering Research Centre for Fruits and Vegetables Processing, Ministry of Education, China Agricultural University, Beijing, China

**Keywords:** spore germination, supernatants, germination receptor, pressure and temperature, spore inactivation

## Abstract

Spores from the *Bacillus* species pose a challenge to the food industry because of their ubiquitous nature and extreme resistance. Accumulated evidence indicates that it is effective to induce spore germination homogenously before killing them. However, it is difficult to obtain and apply exogenous germination factors, which will affect food composition. Therefore, this study screened endogenous germinants from microorganisms by assessing the effect of *Escherichia coli*, *Bacillus subtilis*, *Saccharomyces cerevisiae*, *Lactiplantibacillus plantarum*, and *Streptococcus thermophilus* cultures (cell-free) on *B. subtilis* spore germination. The results showed that the supernatants from these five microorganisms induced spore germination instead of sediments. Moreover, the supernatants of *E. coli*, *B. subtilis*, and *S. cerevisiae* exhibited higher germination rates than *L. plantarum* and *S. thermophilus*, and the induction effects were concentration-dependent. Furthermore, plate counting confirmed that the microbial supernatants induced the lowest spore germination ratio on strains *B. subtilis* FB85 [germination receptors (GRs) mutant] but not strains *B. subtilis* PB705 (PrkC mutant). In addition, *B. subtilis* and *S. cerevisiae* supernatants, combined with pressure and temperature, were effective in spore inactivation. The findings suggested that microbial supernatants may include agents that induce spore germination and may be used for spore inactivation.

## Highlights

- Supernatants, not sediments, from several bacteria could induce spore germination.

- Bacterial supernatants could induce spore germination through GRs and PrkC signaling pathways.

- Bacterial supernatants could be used to develop a germination agent, which could inactivate spore effectively combined with pressure and temperature.

## Introduction

The *Bacillus* species is a major concern in the food industry as its spores can trigger food spoilage and even food poisoning (Kochan et al., 2018; Ortiz et al., 2019). Accumulated evidence indicates that microorganisms, especially spore-producing bacterium, contribute to more than 70% of food poisoning incidents (Ishaq et al., 2021). Therefore, it is urgent to search for efficient, simple, and rapid methods to control spores in food processing industries in particular, as their eradication from foodstuffs may be difficult. Previous studies showed that spores reduced resistance to various treatments and were relatively easily killed during germination (Drusano et al., 2009). Thus, spore germination has been found to be the key step to spore inactivation.

The germination pathways of most spores have been mainly divided into two types: nutrient and non-nutrient germinants (Setlow et al., 2017). In the former, various nutrient germinants, which commonly include sugars, amino acids, inorganic salts, purine nucleosides, or combinations of these molecules, pass through the outer layers of spores and interact with germinant receptors (GRs) located in the inner member of spores, such as GerA in response to L-alanine or L-valine and GerB and GerK in response to the combination of L-Asparagine, D-Glucose, D-Fructose, and potassium ions (K^+^; AGFK; Grela et al., 2018). In contrast, “non-nutrient” germinants, which may be chemical (Ca^2+^-dipicolinic acid, CaDPA), enzymic (lysozyme), or physical (pressure), induce spore germination in a receptor-independent process (Popham et al., 1999; Paidhungat et al., 2001, 2002; Perez-Valdespino et al., 2013). In addition, fragments of vegetative cell peptidoglycans (PGNs) induce spore germination by activating a protein kinase. PGNs can interact with the PASTA (penicillin binding-associated and serine/threonine kinase-associated) domain of PrkC kinase, and activated PrkC induces spore germination by delivering the signal to downstream proteins such as phosphorylating EF-G (Shah et al., 2008). However, most of these germinants are not used in food processing because it is difficult to gain substances such as peptidoglycans in large amounts. Therefore, it is of great significance to screen the germinants for inducing spore germination, and then sterilize them to effectively control food spoilage.

Previous studies showed that various bacteriocins derived from microorganisms could induce or inhibit the germination and outgrowth of different kinds of spores. For example, cell-free supernatants, derived from Gram-positive and Gram-negative species, could promote spore germination, mainly because of peptidoglycans (Shah et al., 2008). Moreover, various bacteriocins produced by bacteria, including *Lactiplantibacillus plantarum*, *Lactococcus lactis*, and *Streptococcus thermophilus*, have demonstrated a broad range of inhibitory activities against spores (Mathot et al., 2003). Bacteriocins including nisin could inhibit the outgrowth of the *Clostridium difficile* germinating spore (Nerandzic and Donskey, 2013; Le Lay et al., 2016). In addition, nisin A, produced by *L. lactis* was found to reduce spore viability by 40–50% (Le Lay et al., 2016; Chai et al., 2017). In addition, its combination with pressure or other chemicals could strengthen the sensitization of spores (Modugno et al., 2019). Furthermore, bacteriocins have been developed as probiotic candidates owing to their antimicrobial potential.

Therefore, to identify more efficient germinants for spore germination that can be used to kill spores, this study comprehensively investigated the effects of cultural compounds derived from various bacteria. Utilizing the *Bacillus subtilis* spore as a model, *Escherichia coli*, *B. subtilis*, *Saccharomyces cerevisiae*, *L. plantarum*, and *S. thermophilus* were selected, and their cell-free supernatants and sediments were functionally analyzed. Furthermore, the pathway of *B. subtilis* spore germination was assessed using mutants. Finally, the effect of the supernatant on killing spores was analyzed by combining it with pressure and temperature. The results of this study can provide new ideas and methods for the inactivation of spores, which is of great significance to ensuring food safety.

## Materials and Methods

### The Preparation of *Bacillus subtilis* Strains and Spore

All *B. subtilis* strains used in this study were derivatives from the strain 168 (China General Microbiological Culture Collection Center, CCGMC). The wild-type strain is the *B. subtilis* 168, and strains *B. subtilis* FB85 (GRs mutant) and PB705 (PrkC) were obtained from Peter Setlow, Department of Molecular, Microbial and Structural Biology, University of Connecticut Health Center, Farmington, CT 06030-3,305 United States. *Bacillus subtilis* spores of various strains were routinely prepared as follows.

A fresh single colony grown on a Luria Bertani (LB) plate was selected and diluted into a moderate LB broth medium at 37°C and 200 rpm until OD_600_ = 0.5. Then, the vegetative cell of *B. subtilis* was obtained. The vegetative cell was inoculated into 2 L Difco Sporulation Medium (DSM) for a further 20–24 h at 37°C and 200 rpm. When >90% of cultures were deemed free spores *via* phase-contrast microscopy, the culture was centrifuged for 15 min at 8,000 *g*, and the supernatant was carefully removed. The spores were kept at 4°C overnight and cleaned with cold distilled water and a histodenz gradient several times through repeated centrifugation. Final spore suspensions were determined by phase-contrast microscopy and stored in 4 or −80°C.

### Preparation of Supernatants and Sediments

**Figure 1:**
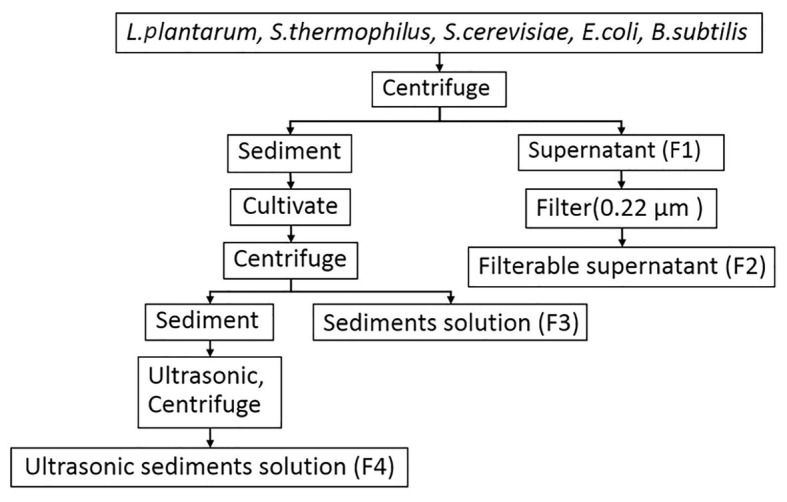
The flow diagram for the treatment of the supernatant and sediment from microorganisms used in this study.

*Escherichia coli* o157, *B. subtilis* 168, *S. cerevisiae* ATCC 9763, *S. thermophilus* CICC 6220, and *L. plantarum* CICC 20265 were used as the experimental materials. *Escherichia coli* and *B. subtilis* strains were cultured in LB broth medium at 37°C for 24 h. *Saccharomyces cerevisiae* was cultured in Yeast Extract Peptone Dextrose medium (YPD) at 30°C for 24 h. *Streptococcus thermophilus* and *L. plantarum* were cultured in de Man Rogosa and Sharp medium (MRS) at 37°C for 14 h. When the OD_600_ of the full-concentration broth was 3.0, the solution was collected and centrifuged. Then, the supernatants (F1) of various microorganisms were filtered with sterilized 0.22-μm millipore filters (Millipore, United States), and this was named the filterable supernatant (F2). The sediments were then cultivated on a plate for 12 h, resuspended with sterile water, and centrifuged, and the supernatant was named the sediment solution (F3). Then, the sediments were broken with an ultrasonic cell disruptor (at 400 Hz, 10 cycles, 10 s interval) and centrifuged, and the supernatant was named the ultrasonic sediment solution (F4; Figure 1). To avoid the detrimental effects of acidic substances produced in the cell culture process, the sediment and supernatant were concentrated by centrifugation and diluted with PBS at pH 7.0–8.0 for subsequent experiments. In addition, the different media were processed as a supernatant and detected in experiments to exclude the potential impact of nutrients from the culture medium (data not shown).

### Induction Activity of the Supernatants

Cell-free filterable supernatants (F2) were prepared as described above (Materials and Methods Section “Preparation of Supernatants and Sediments”). When the OD_600_ value of bacterial suspensions was 3.0, the concentration of cell-free filterable supernatants was determined to be 1. Then, the concentration of the supernatant was diluted to 0.8, 0.6, 0.4, 0.2, 10^−1^, 10^−2^, and 10^−3^ times of the initial bacterial solution (OD_600_ = 3.0), respectively. Supernatants with different concentrations were diluted in sterile water. To completely clear the state of the spore, approximately 150 μl of the supernatants were added to each well that contained spores, and the assessment indexes were detected. In the control group, equal volumes of sterile water were added, and 10 mM AGFK or L-valine was added into the positive groups. When spores were cultivated at 37°C for 3 h, the levels of the dipicolinic acid (DPA) release and OD_600_ were Determined Every 5 min. After incubation, the count plates were performed and assessed.

### DPA Detection

The spore germination experiment with nutrients was performed by using the DPA detection method described by Hindle and Hall (1999), with some adjustments. Spores with an initial OD_600_ value of 0.5 were used in the germination experiment at 37°C in 200 μK-HEPES buffer (25 mM, pH 7.4) containing TbCl_3_ (50 μM). After adding the different germinants, the spore suspensions were cultured at 37°C. The Tb-DPA fluorescence was determined every 5 min for 100–180 min and expressed as relative fluorescence units (RFU) through a multi-well fluorescence plate reader TECAN Spark 10 M. The excitation and emission wavelengths were 270 and 545 nm, respectively. The DPA release ratio was calculated by using this formula: DPA % = (F_1_−F_0_)/(F_2_−F_0_) [F_0_, F_1,_ and F_2_ represent the fluorescence value of the control, treated, and positive (121°C/20 min) groups, respectively].

### OD_600_ Detection

The OD_600_ was measured by the TECAN Spark 10 M by using the measurement of absorbance. Then, 200 μl of spore suspension was transported in the transparent corning 96-well plate, and the value at 600 nm was collected. The fractional ratio of OD_600_ value represents the germination of spores, and the formula used is as follows: germination rate = OD_t_/OD_0_ (OD_0_, OD_t_ represent the absorbance value of initial time and measurement time groups, respectively). Moreover, the decrease in the ratio indicates spore germination, and the increase in the ratio signifies spore outgrowth. The detection was duplicated three times.

### Cell Counting

The *B. subtilis* spore counts were performed through pour-plate enumeration three times. One milliliter of diluted spore suspension was inoculated into each plate and incubated aerobically at 37°C for 24 h. In each experiment, the spore counts of untreated samples represented the initial concentration, which was adjusted to approximately 10^8^ spores/ml *via* a plate count. The logarithm of survivors [log_10_ (N_0_/N_t_)] could indicate the number of reduced spores after different treatments. The spore counts before and after heating at 80°C for 20 min were marked as N_0_ and N_t_, respectively.

### The Treatment With Pressure and Temperature

On the sterile operating table, the spore suspension was adjusted with sterile deionized water, AGFK, L-val, and supernatants from *B. subtilis* or *S. cerevisiae* to OD_600_ = 0.5. L-val, AGFK, and the untreated groups were used as the positive and negative control, respectively. Then, 2 ml of mixed samples were packed in sterilized nylon/polyethylene composite cooking bags (4 cm * 4 cm). These bags were treated with 90°C for 10 min and marked as heat shock treatment (90°C/10 min). The pressure treatment conditions were 200 MPa with a holding time of 20 and 30 min at 80°C, which was followed with heat shock treatment. After treatment with pressure and temperature, spore suspension was performed with cell counting.

### Statistical Analysis

Data are expressed as the mean value ± SD of triplicates. Statistical analysis was determined by using the *t*-test, and differences with a value of *p* < 0.05 were considered statistically significant.

## Results

### The Effect of Endogenous Materials Derived From Different Microorganisms on Spores

**Figure 2:**
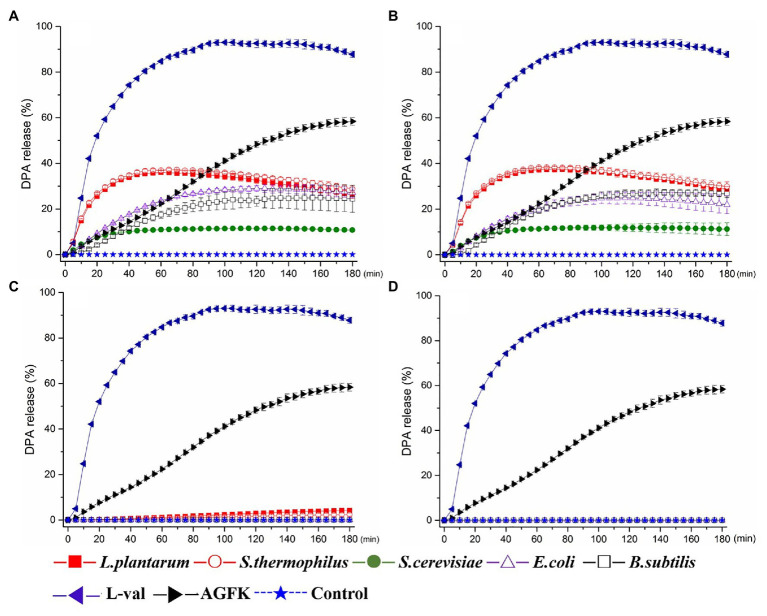
Dipicolinic acid (DPA) release of wild-type *Bacillus subtilis* 168 spores with the treatments of various solutions from *Escherichia coli*, *B. subtilis*, *Saccharomyces cerevisiae*, *Lactiplantibacillus plantarum*, and *Streptococcus thermophiles*. **(A)** The treatment of supernatant from five microorganisms, respectively. **(B)** The treatment of filterable supernatant from five microorganisms, respectively. **(C)** The treatment of sediments solution from five microorganisms, respectively. **(D)** The treatment of ultrasonic sediments solution from five microorganisms, respectively. The treatments of L-val and AGFK were used as the positive control, and untreated spore were control.

To screen spore germinants, we performed germination experiments using *B. subtilis* spores and five different cell-free supernatants. The supernatants, filtered supernatants, solid and liquid bacterial precipitation after ultrasonic crushing from *B. subtilis*, *E. coli*, *S. thermophilus*, *L. plantarum*, and *S. cerevisiae* were prepared and exposed to 10^8^ CFU/ml spores of *B. subtilis* strain 168 at 37°C. Then, the levels of DPA release were determined, and the ratio of DPA release was calculated to determine their effect on spore germination. For comparison, *B. subtilis* spores were treated with L-val and AGFK. These results showed that exposure of 168 spores to L-val induced abundant DPA release during spore germination, with a 90% DPA release ratio. In the experiments with the treatment of supernatants, *S. thermophilus* and *L. plantarum* exhibited the same effect for DPA release of spores, which was better than the others, with a 30–40% ratio. The DPA release ratio of spores with *B. subtilis* and *E. coli* supernatants was 20–30%, although the supernatants from *S. cerevisiae* could induce a 10% DPA release ratio (Figure 2A). Interestingly, the filtered supernatants from five microorganisms showed the same induction effect on spore germination (Figure 2B), but the sediment solution and ultrasonic sediment supernatant did not show any induction effect for the DPA release of spores (Figures 2C,D).

**Figure 3:**
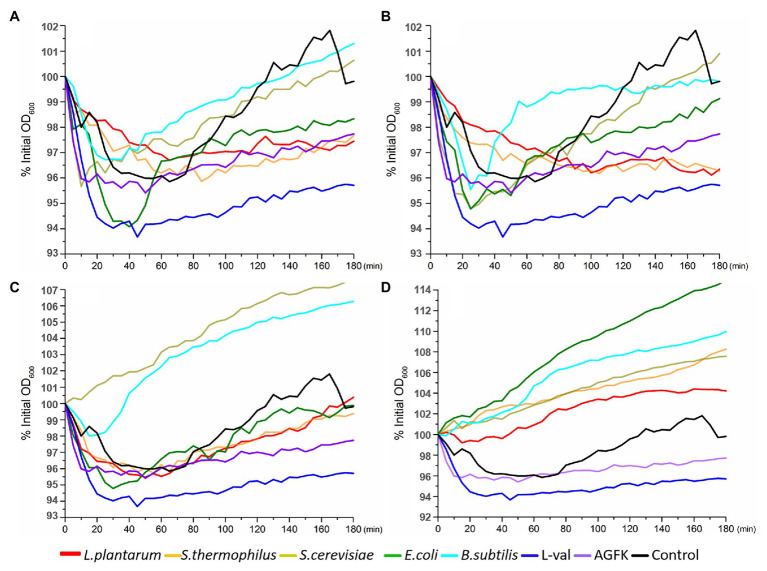
OD_600_ detection of wild-type *B. subtilis* 168 spores with the treatments of various solutions from *E. coli*, *B. subtilis*, *S. cerevisiae*, *L. plantarum*, and *S. thermophiles*. **(A)** The treatment of supernatant from five microorganisms, respectively. **(B)** The treatment of filterable supernatant from five microorganisms, respectively. **(C)** The treatment of sediments solution from five microorganisms, respectively. **(D)** The treatment of ultrasonic sediments solution from five microorganisms, respectively. The treatments of L-val and AGFK were used as the positive control, and untreated spores were control.

The optical density of the spore solution has been used as the assessment index for spore germination, and a decreasing OD_600_ indicates spore germination (Moir and Smith, 1990). Therefore, the absorbance at 600 nm every 10 min for 3 h was detected. Compared to the control, the OD_600_ of spore solution showed an increasing trend after an initial decrease with the treatment of supernatants (F1) and filtered supernatants (F2), but not the sediments solution (F3) and sediment supernatant (F4; Figures 3A–D). Furthermore, the decrease in OD_600_ was found in the first 40 min. These results suggested that the supernatants from *B. subtilis*, *E. coli*, *S. cerevisiae*, *S. thermophilus*, and *L. plantarum* may exhibit the benefits of inducing spore germination but not sediments.

### The Effect of Microorganism Supernatants on Inducing Spore Germination

**Figure 4:**
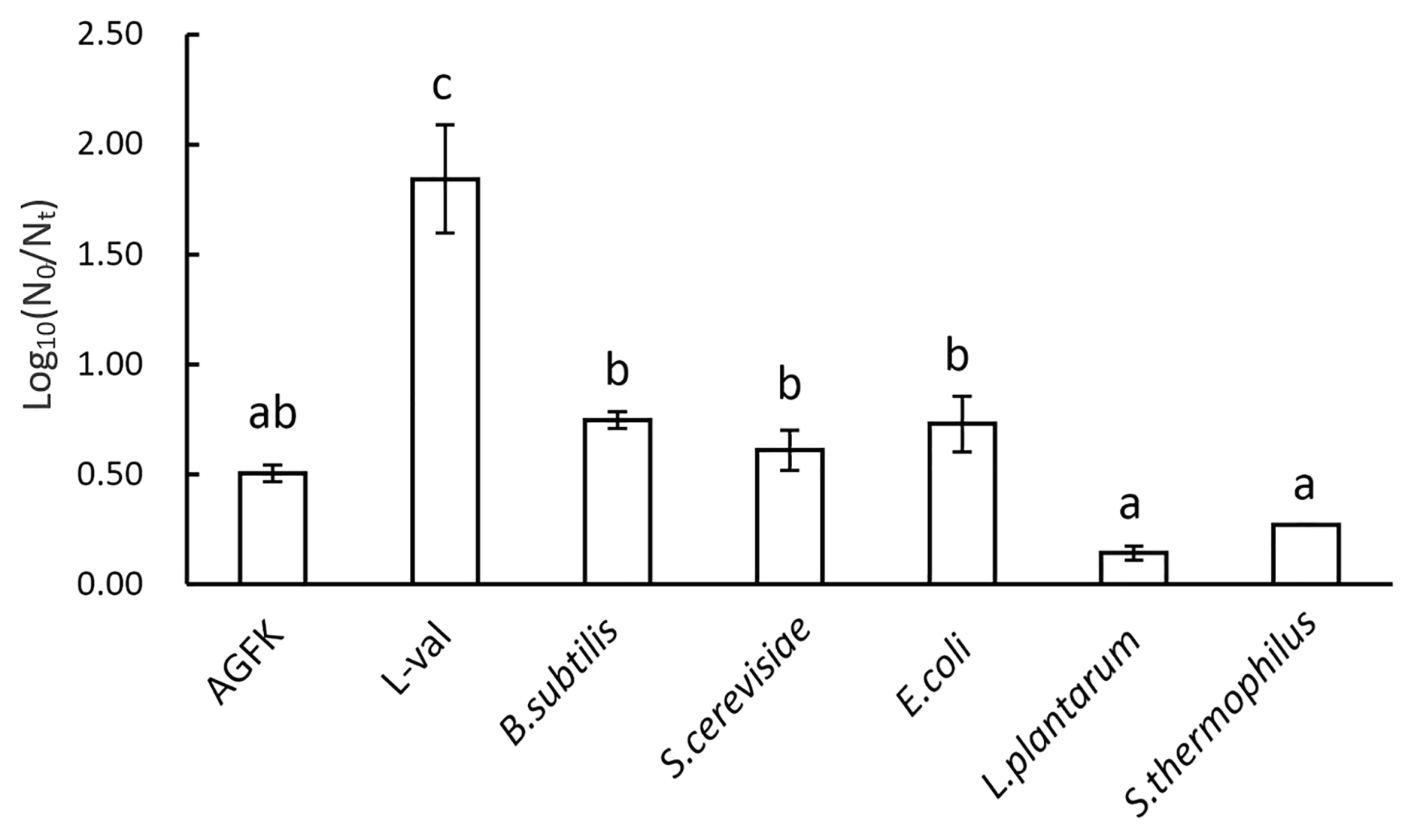
The germination rate of *B. subtilis* 168 spores with various nutrient germinants or microbial supernatants.

To explore the influence of bacterial supernatants on spore germination and outgrowth, we exposed 10^8^ spores/ml of the *B. subtilis* 168 to L-val, AGFK, and five supernatants F2 at 37°C for 3 h and determined the level of germination by plate counting. Compared to the control, the *B. subtilis* 168 was treated using the same conditions but without the germinants. The exposure of spores to L-val could induce germination at a maximum rate of 2.0 log. Spores treated with the *B. subtilis*, *E. coli*, and *S. cerevisiae* supernatants displayed the same germination rate, close to 1.0 log, which was lower than that of the L-val treatment and the same as that of the AGFK treatment. However, spores treated with *S. thermophilus* and *L. plantarum* supernatants showed the lowest spore germination rate (about 0.2 log; Figure 4). All of these suggested that the supernatants from different microorganisms exhibited different effects, and *B. subtilis*, *E. coli*, and *S. cerevisiae* could derive the substance that exhibited better induction effects for spore germination.

### The Relationship Between the Concentration of Supernatant and Spore Germination

**Figure 5:**
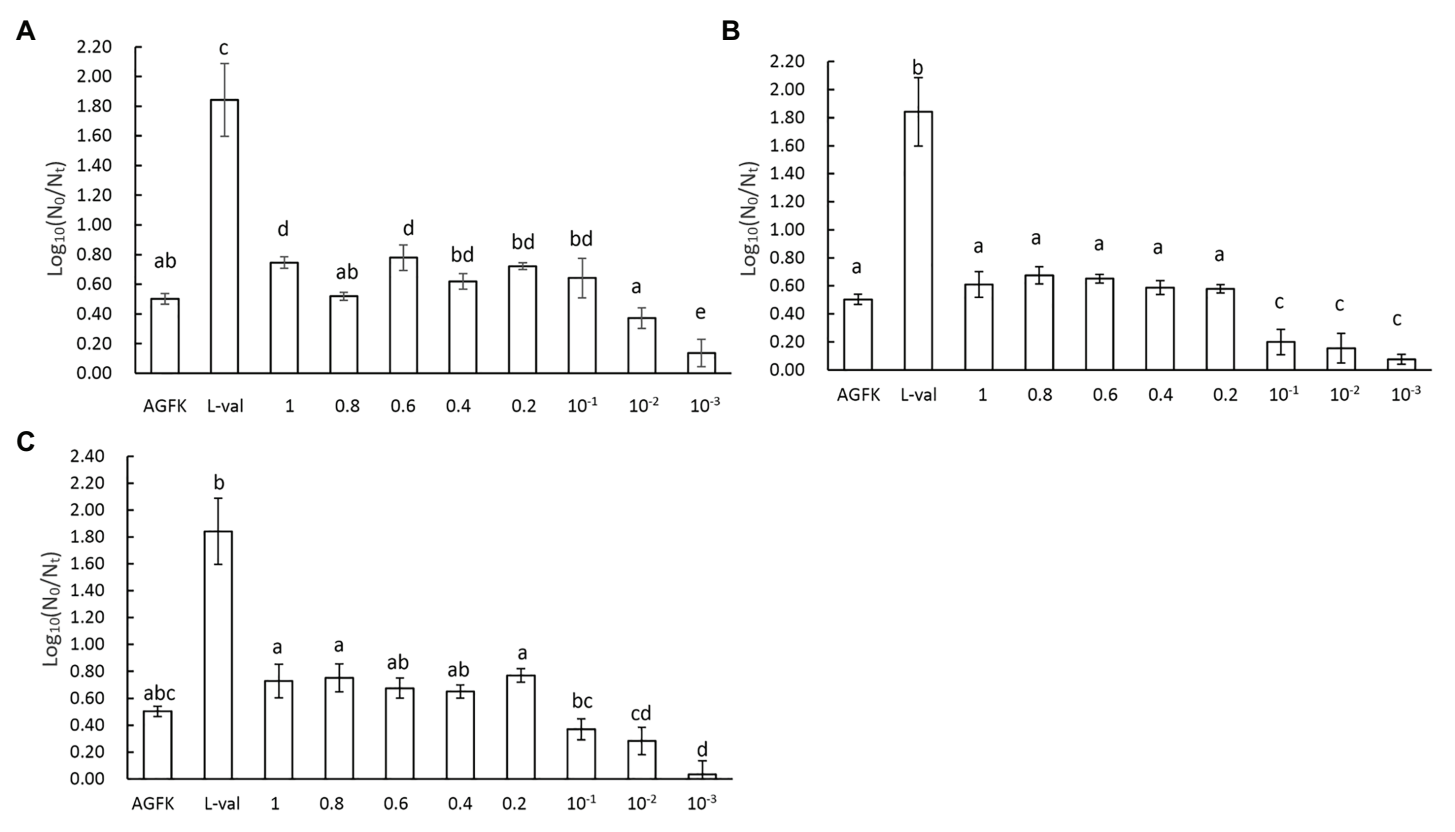
The concentration effect of microbial supernatants on spore germination. **(A)** The treatment of spores with supernatant from *B. subtilis*, **(B)** the treatment of spores with supernatant from *E. coli*, and **(C)** the treatment of spores with supernatant from *S. cerevisiae*. The OD_600_ value of the initial bacterial suspension was 3, the supernatants from which were marked as 1. After diluting 0.8, 0.6, 0.4, 0.2, 10^−1^, 10^−2^, and 10^−3^ times, the supernatants were 0.8, 0.6, 0.4, 0.2, 10^−1^, 10^−2^, and 10^−3^, respectively.

As we focused on the effect of supernatants, different concentrations of microbial supernatants derived from *B. subtilis*, *E. coli*, and *S. cerevisiae* were prepared and used to induce spore germination. The results showed that the *B. subtilis* supernatants at concentrations within the range of 0.1–1.0 (1.0 represents the OD_600_ = 3, 0.1 was 10 times the dilution of supernatants with OD_600_ = 3) induced the germination of 0.7 log spore. When the concentration was <0.1, spore germination decreased (Figure 5A). Moreover, the supernatants from *E. coli* and *S. cerevisiae* exhibited a consistent effect on spore germination, with a 0.7 log (Figures 5B,C). These suggested that three kinds of supernatants with concentrations between 0.1 and 1 exhibited the same effect, whereas concentrations <0.1 inhibited the germination effect (Figures 5A–C). Therefore, these results revealed that three kinds of supernatants from *B. subtilis*, *E. coli*, and *S. cerevisiae* could induce spore germination in a concentration-dependent manner.

### The Functional Pathway of Supernatants in Inducing Spore Germination

**Figure 6:**
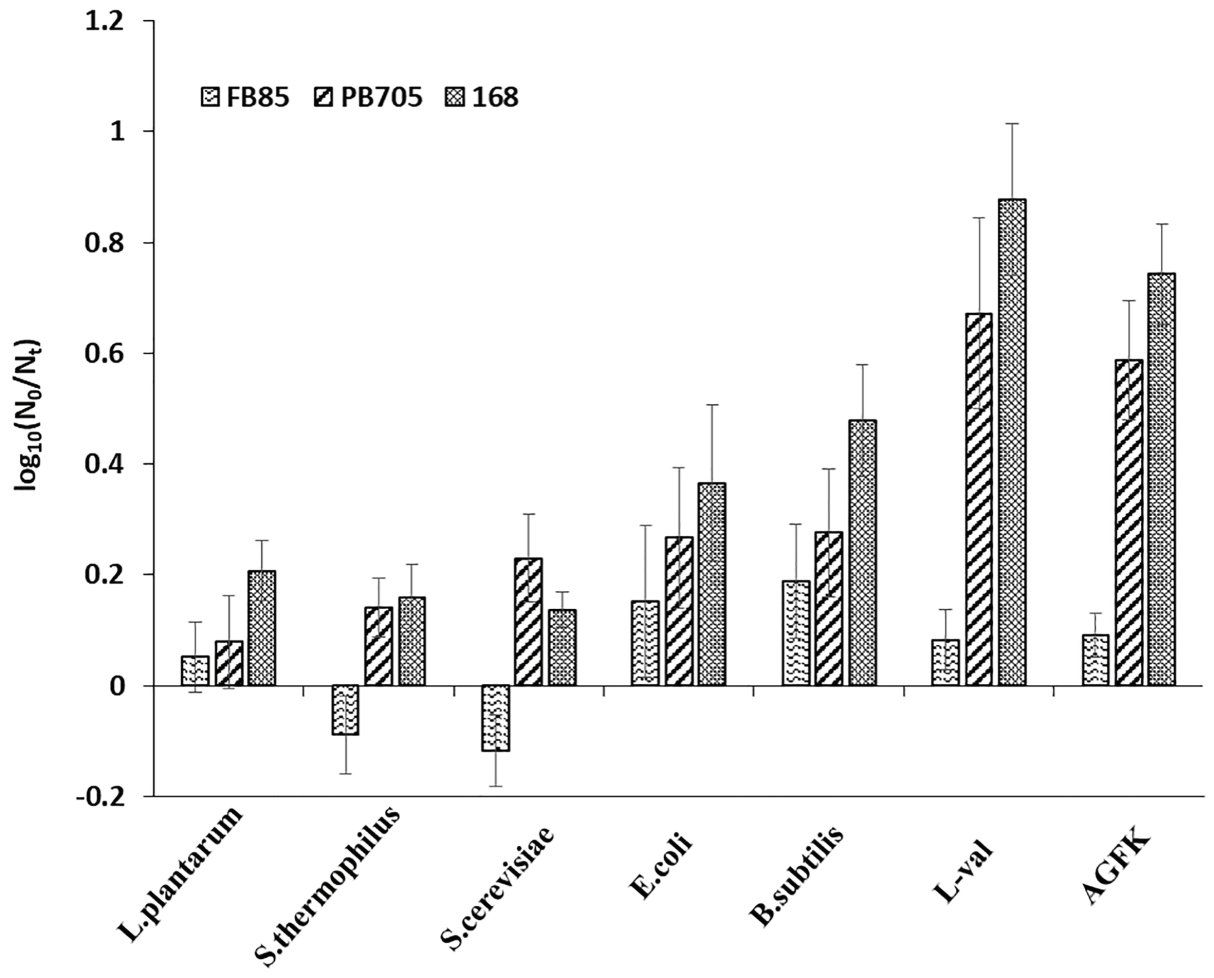
The germination of *B. subtilis* 168, FB85, and PB705 spores with five microbial supernatants and two known germinants (L-val and AGFK). *Bacillus subtilis* FB85 was germination receptors (GRs) mutant strain, and *B. subtilis* PB705 was PrkC mutant.

The previous experiments demonstrated that the substance of supernatants exhibited the effect of inducing spore germination. To further explore the underlying mechanism of supernatants in inducing spore germination, we performed the plate count experiments using GRs or PrkC mutant strains. The supernatants from *B. subtilis*, *E. coli*, and *S. cerevisiae* were used to treat spores, and the germination rates were determined. The results showed that the germination rate of the FB85 spores (GRs mutant) was the lowest (~0.15–0.2), and that of PB705 spores (PrkC mutant) was intermediate (0.1–0.3), although less than 168 spores (0.2–0.5; Figure 6). Moreover, the DPA release experiment showed the same result (data not shown). Therefore, we hypothesize that some substances in supernatants can induce spore germination, mainly through activating the GRs.

### Effect of Pressure and Temperature Combined With Supernatants on Spore Germination

**Figure 7:**
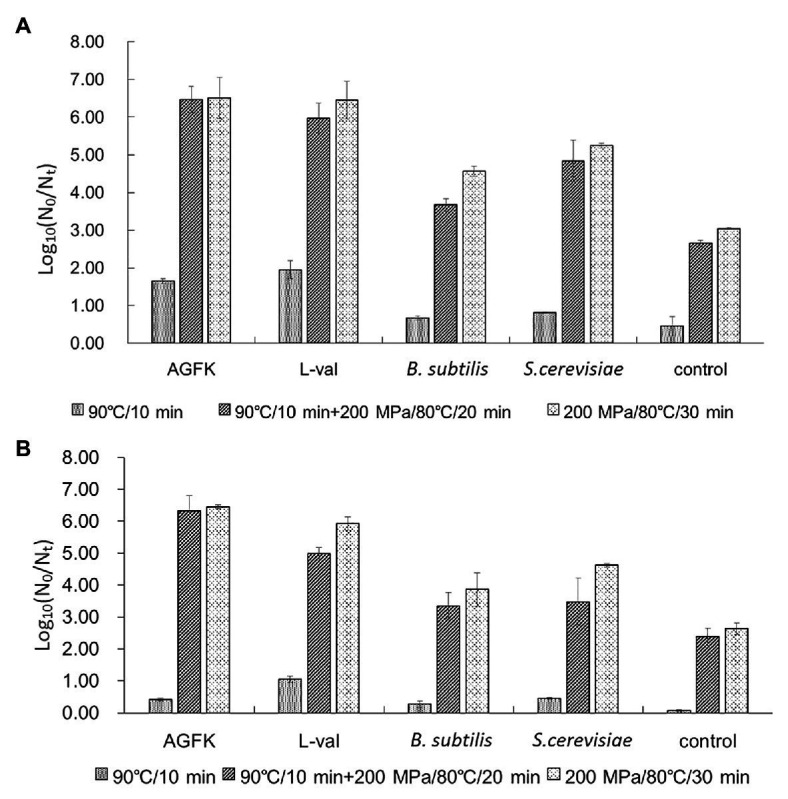
The effect of supernatants from *B. subtilis* and *S. cerevisiae* on spore germination and inactivation. **(A)** The germination of *B. subtilis* 168 spores with different treatments. **(B)** The inactivation of *B. subtilis* 168 spores with different treatments. The supernatants from *B. subtilis* and *S. cerevisiae* combined with temperature and pressure were performed simultaneously.

To further explore the application of bacterial supernatants in food sterilization, we analyzed the combination of temperature and pressure on spore germination and inactivation. First, the combination analysis of heat-activated (90°C/10 min) treatment and various germinating agents showed that, as shown in Figure 7A, AGFK and L-val could induce 1.65 and 1.95 log spore germination, respectively, while the supernatant of *B. subtilis* and *S. cerevisiae* could induce 0.67 and 0.81 log spores, which were higher than the control group (0.46 log spores). Moreover, with the treatment of 90°C/10 min + 200 MPa/80°C/20 min, 6.47 and 5.98 log spores germinated in the AGFK and L-val groups, which were 4.82 and 4.02 log spores more than the heat-activated spores, respectively. The supernatants of *B. subtilis* and *S. cerevisiae* could effectively increase 3 and 4.03 log spores of germination compared with the heat-activated groups, respectively. Interestingly, the same effective spore germination could also be induced by the synergy of pressure and temperature for a longer time (200 MPa/80°C/30 min), whereas ~1.0 log spore increments were achieved in treatment with supernatants of *B. subtilis*.

In addition, the spore-killing effect under the same conditions was also analyzed. The result (Figure 7B) showed that heat activation treatment combined with AGFK, L-val, and the supernatant of *B. subtilis* or *S. cerevisiae* could kill 0.42, 1.05, 0.27, and 0.45 log spores, which was much higher than that of the control group (0.07 log). The higher inactivation level was observed in two pressure and temperature treatment combinations: 90°C/10 min + 200 MPa/80°C/20 min and 200 MPa/80°C/30 min. For the treatment of *B. subtilis* and *S. cerevisiae* supernatants, the former could kill 3.35 and 3.47 log spores, respectively, which were significantly higher than 2.38 log spores in the control group (*p* > 0.05), and the latter had 3.86 and 4.62 log inactive spores. All of these results suggested that the combination of temperature and pressure treatment can increase the effect of *B. subtilis* and *S. cerevisiae* supernatants in inducing spore germination and reducing spore activation.

## Discussion

In this study, we developed a strategy to alter spore germination through the use of microbial culture products, which could be developed as a kind of food additive in non-thermal sterilization technology. Our data showed that treatment with various microbial supernatants, including *E. coli*, *B. subtilis*, and *S. cerevisiae*, induced spore germination, but the effects of *S. thermophilus* and *L. plantarum* were not distinct. These results suggested that the stimulus from the microbial supernatants, which only exist in some microorganisms, have the potential to induce spore germination. Further investigation indicated that the induction effects of supernatants depend on their concentration, and the effects were not observed at low concentrations (<0.1). These results indicated that the endogenous factors in microbial supernatants may induce spore germination. Moreover, combined with pressure and temperature, *B. subtilis*, and *S. cerevisiae* supernatants exhibited the effect of killing spores. Collectively, our results not only indicated that microbial supernatants may be potentially used in inducing spore germination, but also may be utilized as an effective germination agent in food processing.

Supernatants have the effect of inducing spore germination, but not the sediments. Microorganisms secrete many kinds of antimicrobial substances that inhibit or trigger the growth of bacterial by directly breaking the membrane structure of bacteria, including bacteriocins, organic acids, and some heat-resistant small peptides (Lash et al., 2005; Song et al., 2014). The antimicrobial activity of *Lacticaseibacillus rhamnosus* was exhibited entirely through secreted cell-free supernatants (He et al., 2017). Moreover, these chemical substances were secreted from bacteria only during the index growth period. The cell-free supernatants from *E. coli* and *B. subtilis* could induce the germination of spores through the PGN derived from the cell wall (Shah et al., 2008). Additionally, the bacteria cell wall was ultrasonically decomposed and various big fragments emerged, but PGN was obtained through zymolysis (Desmarais et al., 2014). Our findings suggested that there may be active components in the bacterial supernatant that impart beneficial effects on spore germination. Moreover, the subtilin produced by *B. subtilis* ATCC 6633 may exhibit antimicrobial effects, which belong to a kind of LAB bacteriocins (Lee and Kim, 2011). Thus, we hypothesized that these germinants can be metabolized by microorganisms during the growth period and are not derived from the damaged bacteria, which will be investigated carefully in future research. In addition, the low concentration of supernatants from microbial species used here did not show better induction effects; however, the moderate concentration of cell-free supernatants induced spore germination (Figures 5A–C). When OD_600_ of bacterial suspension was 3.0, the microorganism is in the period of stable growth, and a large number of metabolites were produced in catabolism, including PGN fragments from the cell wall, which may contribute to spore germination. To date, many bacteriocins have been exploited in food additives, including nisin and lacticin (Galvez et al., 2007; Gharsallaoui et al., 2016; Castellano et al., 2018; Sanborn et al., 2018). Therefore, screening and application of spore germinants as food additives need to consider their working concentration and edible dosage.

In addition, the five kinds of supernatants could trigger the DPA release, and the levels of DPA release on spores with *S. thermophilus* and *L. plantarum* were higher than the treatment of *B. subtilis*, *E. coli*, and *S. cerevisiae* (Figure 2). The ratio of DPA release indicated that germination commitment and spore germination would ensue (Yi and Setlow, 2010; Zhang et al., 2014). However, *S. thermophilus* and *L. plantarum* did not significantly improve the levels of germination (Figure 4). Various studies have demonstrated that some substances secreted by *S. thermophilus* and *L. plantarum* could inhibit bacteria growth significantly (Rossi et al., 2013; Sanborn et al., 2018). These findings were consistent with the results that substances derived from *S. thermophilus* could inhibit the germination and outgrowth of *C. difficile* spore (Chai et al., 2017; Setlow et al., 2017). In addition, the effects of various bacteriocins, such as nisin, enterocin AS-48, and thurincin H, were unlikely to occur *via* direct induction of dormant spores, but mainly inhibited the germinating spores (Lee and Kim, 2011). The possible reason was that in the early germination period, substances from *S. thermophilus* and *L. plantarum* cell-free supernatants could establish germination commitment and trigger DPA release, and thus spore germination could not be reversed. Then, during the spore outgrowth period, the process could be inhibited by other substances.

The present study shows that supernatants may be potentially utilized in inducing spore germination. However, the specific component of the supernatant that exerts the beneficial effects remains unclear. Supernatants are complex mixtures containing various substances, including organic acids, lipids, proteins, and other small molecules (Lievin-Le Moal and Servin, 2014). Owing to their complexity and uncertainty, it is difficult to ascertain the multiple effects of supernatants. To date, the PGN derived from bacterial cell walls can interact with PrkC kinase and transduce the signal to trigger spore germination (Shah et al., 2008). In our results, the mutant spore germination experiment revealed that the germinants from supernatants mainly interacted with the GRs because GR mutant spore germination was almost inhibited. However, the PrkC mutant spores exhibited a relatively high germination ratio (Figure 6). Thus, the GRs pathway could play a more important role than the PrkC pathway in establishing the effects of supernatants. Interestingly, the log (N_0_/N_t_) of the GRs mutants was negative with the treatment of *S. thermophilus* and *L. plantarum* cell-free supernatants. A previous study showed that heat shock induced spore germination mainly through GRs (Luu et al., 2015), but it is not clear whether there are other germination pathways. Our results implied that other germination pathways may exist. In subsequent experiments, we will try to explore the details of cell-free supernatants in inducing spore germination (e.g., the substances that play a role in inducing spore germination and their functional mechanism).

At present, non-thermal sterilization technology has been widely examined, particularly in spore inactivation research, because it can better maintain the flavor and color of food (Olivier et al., 2011). Previous study showed that the mechanism of spore inactivation was different with various pressures and temperatures due to different spore germination receptors. Specifically, 200–500 MPa could activate GRs (Doona et al., 2014; Liang et al., 2019), although >500 MPa could activate the channel proteins of DPA release, SpoVA, resulting in spore germination (Reineke et al., 2013). Although pressure treatment alone could reduce the limited number of spores, the time required was also from 5 to 45 min, resulting in high energy consumption (Reddy et al., 2016). Temperature combined with pressure has been useful technology, and the normal range is 80–100°C, but spores cannot completely be killed (Liang et al., 2019). Therefore, the combination of effective germination agents may be more effective in spore inactivation. We combined pressure and temperature with *B. subtilis* and *S. cerevisiae* supernatants that could inactivate an average of ~4 log spores, especially in the treatment of 200 MPa/80°C/30 min.

In summary, our study revealed that microbial supernatants could induce spore germination mainly by activating nutrient receptors, and combined with pressure and temperature, effectively inactivated spores. Thus, we believe that supernatants may be potentially used as an alternative treatment for preventing spore outgrowth or inducing spore germination. The results also confirmed that supernatants from certain microorganisms can exhibit similar beneficial effects as their anti-bacterial counterparts. Therefore, it is necessary to further identify the active ingredients in supernatants to develop novel antibiotics for use in food processing.

## Data Availability Statement

The original contributions presented in the study are included in the article/supplementary material; further inquiries can be directed to the corresponding author.

## Author Contributions

LD, FC, and XH conceived and designed the experiments. JL and YS performed all the experiments. LD, FC, XH, JL, and YS discussed the results and drafted and revised the manuscript. All authors contributed to the article and approved the submitted version.

### Conflict of Interest

The authors declare that the research was conducted in the absence of any commercial or financial relationships that could be construed as a potential conflict of interest.
